# A three-domain copper-nitrite reductase with a unique sensing loop

**DOI:** 10.1107/S2052252519000241

**Published:** 2019-02-09

**Authors:** Diederik Johannes Opperman, Daniel Horacio Murgida, Sergio Daniel Dalosto, Carlos Dante Brondino, Felix Martín Ferroni

**Affiliations:** a Department of Biotechnology, University of the Free State, 205 Nelson Mandela Drive, Bloemfontein, Free State 9300, South Africa; b Departamento de Química Inorgánica, Analítica y Química Física and INQUIMAE (CONICET-UBA), Facultad de Ciencias Exactas y Naturales, Universidad de Buenos Aires, Ciudad Universitaria, Pab. 2 piso 1, Buenos Aires, Buenos Aires C1428EHA, Argentina; c Instituto de Física del Litoral, CONICET-UNL, Güemes 3450, Santa Fe, Santa Fe S3000ZAA, Argentina; d Departamento de Física, Facultad de Bioquímica y Ciencias Biológicas, Universidad Nacional del Litoral (UNL), CONICET, Ciudad Universitaria, Paraje El Pozo, Santa Fe, Santa Fe S3000ZAA, Argentina

**Keywords:** *Thermus scotoductus* SA-01, three-domain copper-nitrite reductase, X-ray crystal structure, Ser_CAT_ residue, sensing loop

## Abstract

Dissimilatory nitrite reductases are key enzymes in the denitrification pathway, reducing nitrite and leading to the production of gaseous products (NO, N_2_O and N_2_). The reaction is catalysed either by a Cu-containing nitrite reductase (NirK) or by a cytochrome *cd*_1_ nitrite reductase (NirS), as the simultaneous presence of the two enzymes has never been detected in the same microorganism. The thermophilic bacterium *Thermus scotoductus* SA-01 is an exception to this rule, harbouring both genes within a denitrification cluster, which encodes for an atypical NirK. The crystal structure of *Ts*NirK has been determined at 1.63 Å resolution. *Ts*NirK is a homotrimer with subunits of 451 residues that contain three copper atoms each. The N-terminal region possesses a type 2 Cu (T2Cu) and a type 1 Cu (T1Cu_N_) while the C-terminus contains an extra type 1 Cu (T1Cu_C_) bound within a cupredoxin motif. T1Cu_N_ shows an unusual Cu atom coordination (His_2_–Cys–Gln) compared with T1Cu observed in NirKs reported so far (His_2_–Cys–Met). T1Cu_C_ is buried at ∼5 Å from the molecular surface and located ∼14.1 Å away from T1Cu_N_; T1Cu_N_ and T2Cu are ∼12.6 Å apart. All these distances are compatible with an electron-transfer process T1Cu_C_ → T1Cu_N_ → T2Cu. T1Cu_N_ and T2Cu are connected by a typical Cys–His bridge and an unexpected sensing loop which harbours a Ser_CAT_ residue close to T2Cu, suggesting an alternative nitrite-reduction mechanism in these enzymes. Biophysicochemical and functional features of *Ts*NirK are discussed on the basis of X-ray crystallography, electron paramagnetic resonance, resonance Raman and kinetic experiments.

## Abstract 2

TsNirK is the first three-copper three-domain nitrite reductase harbouring Gln-coordinated type 1 copper centres. All the structural properties of TsNirK point to an enzyme that, despite having several of the essential catalytic features present in other NirKs, shows two distinctive and unique characteristics: firstly, the putative T1CuC → T1CuN → T2Cu electron-transfer pathway along the same subunit; and secondly, more importantly, is the presence of the Ser_CAT_ residue at the enzyme substrate sensing loop, which opens a new paradigm in this widely studied family of enzymes.

## 1. Introduction   

The global nitrogen cycle maintained by some bacteria impacts all forms of life worldwide (Zumft, 1997 ▸; Gruber & Galloway, 2008 ▸; Fowler *et al.*, 2014 ▸). The biological fixation of atmospheric dinitrogen to produce NH_3_ is the process that introduces inorganic nitrogen into the biosphere, while the denitrification process proceeds in the opposite direction. Bacteria convert inorganic nitrogen into organic nitrogen sources by assimilatory pathways during the interconversion of NH_3_, NO_3_^−^ and NO_2_^−^. Dissimilatory denitrification produces dinitrogen by the reduction of NO_3_^−^ and NO_2_^−^, with NO and N_2_O as intermediaries involving several enzymes in the process.

Reduction of NO_2_^−^ to NO (NO_2_^−^ + 2 H^+^ + e^−^ → NO + H_2_O), catalysed by nitrite reductase (Nir), is the key reaction that initiates the dissimilatory denitrification process in denitrifiers (Zumft, 1997 ▸). Two kinds of Nirs are involved in this catalytic step, the haem- and copper-containing enzymes, NirS and NirK, respectively. It was postulated that all denitrifying bacteria harbour only one kind of Nir (Zumft, 1997 ▸). However, this rule has changed since the genomes of *Thermus scotoductus* SA-01 (Gounder *et al.*, 2011 ▸), *T. oshimai* JL-2 (Murugapiran *et al.*, 2013 ▸), and *Bradyrhizobium oligotrophicum* S58 (Okubo *et al.*, 2013 ▸) have been reported, as they carry genes for both NirS and NirK enzymes.

Most NirKs are homotrimers in which each subunit (∼37 kDa) is composed of two domains (Zumft, 1997 ▸; Horrell *et al.*, 2017 ▸), with homologous structural features conserved between NirKs. Each monomer is composed of two consecutive Greek key β-barrel folding domains harbouring one type 1 (T1Cu) and one type 2 copper (T2Cu) centre (Adman *et al.*, 1995 ▸; Nojiri, 2017 ▸). T2Cu is the catalytic active site found at the intersection of two adjacent subunits; two His residues from the same monomer, one His from an adjacent subunit and one water molecule coordinate the copper atom (Adman *et al.*, 1995 ▸; Nojiri, 2017 ▸). T1Cu is an electron-transfer centre which is coordinated to two His, one Cys and one Met residue. On the basis of the UV–Vis spectroscopic features of T1Cu, two-domain NirKs have been classified as class I and class II, or blue and green, respectively (Zumft, 1997 ▸; Merkle & Lehnert, 2012 ▸). T1Cu and T2Cu are ∼12.6 Å apart and linked by a Cys−His bridge that is the proposed electron-transfer pathway that delivers the one electron necessary for reduction of NO_2_^−^ at T2Cu. Both copper centres are also linked by a chemical path longer than the Cys–His bridge, named the sensing loop, which is proposed to trigger the T1Cu–T2Cu electron delivery when nitrite is bound to T2Cu (Strange *et al.*, 1999 ▸). The sensing loop of all NirKs reported so far harbours a conserved Asp residue essential for catalysis, called Asp_CAT_ (Boulanger *et al.*, 2000 ▸; Hough *et al.*, 2005 ▸; Kataoka *et al.*, 2000 ▸).

Class I and class II are the best characterized NirKs. Recently, class III NirKs emerged as three-domain NirKs having, in addition to the two-domain core, an extra haem- or T1Cu-domain fused at the N- or C-terminal region (Antonyuk *et al.*, 2013 ▸; Ellis *et al.*, 2007 ▸; Nojiri *et al.*, 2007 ▸; Tsuda *et al.*, 2013 ▸). To date, three members of class III NirKs have been reported. NirK from *Hyphomicrobium denitrificans* A3151 (*Hd*Nir) shows all the structural features of the two-domain NirK enzymes with an additional N-terminally fused cupredoxin domain containing a T1Cu centre (Nojiri *et al.*, 2007 ▸). However, the extra T1Cu centre is too far away from the two-domain core to be considered compatible with the electron-transfer process (Nojiri *et al.*, 2007 ▸). In contrast, the three-domain Nir from *Ralstonia pickettii* (*Rp*Nir), which contains a C-terminal cytochrome *c* domain fused to the two-domain NirK core, is an effective self-electron-transfer system where the donor and acceptor proteins are naturally fused (Antonyuk *et al.*, 2013 ▸). The third example is the NirK from *Pseudo­alteromonas haloplanktis* (*Ph*Nir); this enzyme is a naturally fused type of Nir tethering a cytochrome *c* at the C-terminus fold as a unique trimeric domain-swapped structure (Tsuda *et al.*, 2013 ▸).

Here we describe the crystal structure of the NirK from *T. scotoductus* SA-01 (*Ts*NirK) at a resolution of 1.63 Å together with its biochemical and spectroscopic characterization. This enzyme is a three-domain NirK that shows the T1Cu centre of the two-domain core with a coordination never observed before, a third cupredoxin motif in close proximity to the T1Cu site of the two-domain core and a sensing loop that does not contain the essential Asp_CAT_. We discuss the structural properties of *Ts*NirK in comparison with the best characterized NirKs and the implications on the catalytic mechanism of this novel enzyme.

## 2. Materials and methods   

### 2.1. Protein sequence analysis and alignment   

The sequences were identified using the *BLAST* (Altschul *et al.*, 1990 ▸) and *FASTA* (Lipman & Pearson, 1985 ▸) webtools. The protein sequences of *Ts*NirK and *Hd*Nir were used as initial search models for the three-domain NirKs. Two-domain NirKs were identified using *Ax*Nir and *Af*Nir as the search models. The UniProt database was searched using the default matrix BLOSUM62.

Sequence alignments were carried out using *MEGA7* (Kumar *et al.*, 2016 ▸), with visualization in *Geneious* 7.0 (https://www.geneious.com/). The evolutionary history was inferred by using the maximum-likelihood method based on the Whelan and Goldman model (Whelan & Goldman, 2001 ▸). The bootstrap consensus tree inferred from 500 replicates is taken to represent the evolutionary history of the taxa analyzed (Felsenstein, 1985 ▸). Branches corresponding to partitions reproduced in less than 50% bootstrap replicates are collapsed. Initial tree(s) for the heuristic search were obtained automatically by applying *Neighbour-Join* and *BioNJ* algorithms to a matrix of pairwise distances estimated using a JTT model and then selecting the topology with superior log-likelihood value. A discrete gamma distribution was used to model evolutionary rate differences amongst sites [five categories (+G, parameter = 2.1930)]. The rate variation model allowed for some sites to be evolutionarily invariable (+I, 3.7109% sites). The analysis involved 37 amino-acid sequences. All positions with less than 95% site coverage were eliminated. Fewer than 5% alignment gaps, missing data and ambiguous bases were allowed at any position. There were a total of 256 positions in the final data set. Evolutionary analyses were conducted in *MEGA7* (Kumar *et al.*, 2016 ▸).

### 2.2. Cloning and overexpression of *Ts*NirK   

A pET-22*b*(+) plasmid containing the *Tsc_c17620* gene (Gounder *et al.*, 2011 ▸) with codon optimization for expression in *Escherichia coli* was purchased from GenScript Inc. The heterologous expression of the *nir*K gene from *T. scotoductus* SA-01 was achieved by transforming pET22:*Ts*NirK into *E. coli* BL21 (DE3) (New England Biolabs Inc.). The recombinant strain was grown aerobically at 37°C overnight with agitation at 200 rev min^−1^ in lysogeny broth with the addition of 100 µg ml^−1^ ampicillin as starter culture. Expression was performed using 400 ml (1/100 starter culture) of auto-induction media (ZYP5052) (Studier, 2005 ▸) plus 100 µg ml^−1^ ampicillin with no lactose addition in 2 l Erlenmeyer flasks maintained at 37°C for 24 h (200 rev min^−1^). CuSO_4_ (200 µ*M*) was added to the high-density culture and maintained in the same condition for 1 h. Finally, the copper-fed culture was induced with 250 µ*M* isopropyl-β-d-1-thio­galactopyranoside (IPTG) at 20°C and 50 rev min^−1^ for 3 h. Cells were harvested through centrifugation and re-suspended in 20 m*M* Tris–HCl (pH 8) buffer. Expression levels were evaluated using SDS–PAGE analysis (Laemmli *et al.*, 1970 ▸) with prestained MRP 2-105 K protein standards (Genbiotech) as molecular mass markers and stained using Coomassie brilliant blue R-250.

### 2.3. Protein purification   

A cell suspension (0.1 g wet weight ml^−1^) was disrupted by sonication. The crude extract was recovered by centrifugation at 25 000*g* for 1 h and dialyzed overnight against 20 m*M* Tris–HCl buffer (pH 8) supplemented with 100 µ*M* CuSO_4_ and again centrifuged at 25 000*g* for 1 h. *Ts*NirK from the crude extract was purified in three chromatographic steps. The crude extract was applied to an anion-exchange column (DEAE Sepharose Fast Flow, 2.6 × 34.5 cm, GE Healthcare) equilibrated in 20 m*M* Tris–HCl buffer, (pH 8) and eluted with 600 ml of a 0–500 m*M* linear gradient of NaCl in equilibration buffer. Deep-blue fractions containing *Ts*NirK were pooled and dialyzed against 20 m*M* Tris–HCl buffer plus 100 µ*M* CuSO_4_. The dialyzed pool was loaded onto a Source 15Q matrix column (1.6 × 13 cm, GE Healthcare) equilibrated with 20 m*M* Tris–HCl (pH 8). Bound proteins were eluted with a linear gradient in equilibration buffer (200 ml; 0 to 600 m*M* NaCl). Finally, fractions with *Ts*NirK were concentrated by an Amicon Ultra 30 K nominal molecular weight limit device and loaded onto a Superdex S200 column (1.5 × 42 cm, GE Healthcare). Fractions (500 µl) were loaded and eluted with 20 m*M* Tris–HCl buffer (pH 8) containing 200 m*M* NaCl. The highly pure turquoise *Ts*NirK fractions were pooled and concentrated to approximately 20 mg ml^−1^ in 20 m*M* Tris–HCl (pH 8) and stored at −80°C. Protein purity was evaluated by SDS–PAGE and followed by UV–Vis spectroscopy through the purification procedure.

### 2.4. Protein content, molecular mass determination and copper content assays   

Protein concentration was determined using the Bradford method with bovine serum albumin as standard (Bradford, 1976 ▸). Spectrophotometric measurements were performed on a Perkin–Elmer Lambda 20 UV–Vis spectrophotometer. The molecular mass of pure enzyme was estimated by gel-filtration chromatography. A prepacked Superdex 200 10/300 G2 column (GE Healthcare) connected to an FPLC device (ÄKTAprime, GE Healthcare) was equilibrated with 150 m*M* Tris–HCl buffer, pH 7.6. Isocratic elution at a flow rate of 0.5 ml min^−1^ was performed with detection at 280 nm. The molecular weight markers used for calibration were ferritin (440 kDa), aldolase (158 kDa), conalbumin (75 kDa), ovalbumin (44 kDa) and carbonic anhydrase (29 kDa), all from GE Healthcare. The molecular mass of the subunits was estimated by SDS–PAGE according to the method of Laemmli *et al.* (1970 ▸). Samples were evaluated on a 10% denaturing polyacrylamide gel after treatment with SDS–PAGE sample buffer for 10 min at 100°C. The prestained mid-range protein marker (2–105 kDa) (Genbiotech) was used to estimate the monomer molecular mass.

The copper content was determined by performing the biquinoline colorimetric method (Klotz & Klotz, 1955 ▸) with modifications. Samples of protein equivalent to 0–50 µ*M* Cu (275 µl) were added of 250 µl biquinoline solution (5 mg ml^−1^ in glacial acetic acid) and 225 µl of 20 m*M* ascorbic acid in phosphate buffer pH 6.0 in sequential order. The reaction mixture was maintained at room temperature for 10 min and the absorbance at 546 nm was measured. Standard calibration curve was obtained performing the procedure on 275 µl standard solutions (0–50 µ*M* CuSO_4_). The copper content in the samples was determined in triplicate.

### 2.5. Activity assays and kinetics   

The nitrite-reducing activity of *Ts*NirK was estimated by standard assay for NirK using methyl viologen as the artificial electron donor (Ferroni *et al.*, 2012 ▸).

In another assay, the reduced form of the pseudoazurin from *Sinorhizobium meliloti* (*Sm*Paz) (Ferroni *et al.*, 2014 ▸) was used as electron donor. A prereaction mixture of 30 m*M* MES–Tris buffer (pH 6.0), 50 µ*M* *Sm*Paz, and 15 n*M* *Ts*NirK in a total volume of 1 ml was maintained in a septa-sealed cuvette under argon flux. To exclude dioxygen, all the solutions were flushed with argon for 30 min. The mixture *Sm*Paz–*Ts*NirK was reduced with sodium dithionite (2.5 µl of 200 m*M* dithionite solution). The reaction was started by the injection of sodium nitrite (10 µl of 100 m*M* solution). The reoxidation of the electron donor was followed at 597 nm. The reoxidation of *Sm*Paz by the action of NirK from *S. meliloti* 2011 (*Sm*Nir) (Ferroni *et al.*, 2014 ▸) was assayed as a positive control. A negative control was performed with no enzyme addition.

### 2.6. Physical measurements   

UV–Vis electronic absorption spectra were recorded on a Perkin–Elmer Lambda 20 UV–Vis spectrophotometer at 298 K. Resonance Raman spectra were acquired at 77 K in a Dilor XY-800 microspectrometer equipped with a Linkam THMS600 freezing microscope stage. Frozen samples were irradiated with 5 mW of a 631.9 nm diode laser (TopMode-633) and the scattered light was collected in backscattering geometry during 2 min at a resolution of 0.4 cm^−1^ per data point. Electron paramagnetic resonance (EPR) measurements were performed at the *X*-band on a Bruker EMXplus spectrometer at 120 K. EPR spectra were simulated with the *EasySpin* toolbox based on *MATLAB* (Stoll & Schweiger, 2006 ▸). Spectra taken in the temperature range 20–200 K showed no significant differences. Samples for EPR spectroscopy were concentrated to ∼200 µ*M* trimeric *Ts*NirK in 20 m*M* MES–Tris buffer (pH 6.0) by an Amicon concentrator. Then 5 µl of 1 *M* degassed stock solutions of sodium dithionite and sodium nitrite were withdrawn by gastight syringe from the vessels containing the respective solutions and loaded into argon-flushed EPR tubes containing samples of *Ts*NirK (∼200 µl) followed by gentle mixing. The EPR tubes were frozen with liquid nitrogen and kept under these conditions until use. The experimental conditions used were: microwave frequency, 9.45 GHz; microwave power, 2 mW.

### 2.7. Crystallization and structure determination   

Sitting-drop vapour-diffusion screening crystallization trials yielded deep-blue *Ts*NirK crystals in several conditions within 2 weeks at 16°C. Single crystals grew in 2 µl drops consisting of equal volumes of 8 mg ml^−1^ *Ts*NirK and reservoir solution [0.2 *M* CaCl_2_·2H_2_O, 0.1 *M* HEPES sodium pH 7.5, 28%(*w*/*v*) PEG 400]. Crystals were soaked in reservoir solution containing 30%(*v*/*v*) glycerol prior to cryocooling. X-ray diffraction data were collected at Diamond Synchrotron (UK) on beamline I04-1 (0.9282 Å) at 93 K. Data were processed using *autoPROC* (Vonrhein *et al.*, 2011 ▸), with indexing and integration using *XDS* (Kabsch, 2010 ▸) and *POINTLESS* (Evans, 2006 ▸), with intensities scaled and merged using *AIMLESS* (Evans & Murshudov, 2013 ▸) from the *CCP*4 suite of programs (Winn *et al.*, 2011 ▸). Molecular replacement was performed using *PHASER* (McCoy *et al.*, 2007 ▸) with *Geo­bacillus thermodenitrificans* Nir (*Gt*Nir, PDB entry 3x1e; Fukuda & Inoue, unpublished work) as the search model. Refinement was carried out through iterative cycles of manual model building in *COOT* (Emsley *et al.*, 2010 ▸) and refinement using *Refmac* (Murshudov *et al.*, 2011 ▸). Structures were validated using programs within the *CCP*4 suite (Winn *et al.*, 2011 ▸). Ramachandran distribution gave 99.5% in the favoured region, with 0.5% in the generously allowed regions. Figures were generated using *UCSF Chimera* (Pettersen *et al.*, 2004 ▸). Tunnels and pocket in the structure were detected using *CASTp* (Dundas *et al.*, 2006 ▸).

Structure factors and model coordinates have been deposited in the Protein Data Bank with the accession number 6hbe.

### 2.8. Computational methods   

A combination of quantum mechanics and molecular mechanics (QM/MM) calculations was used to compute the structure and the Raman spectra of the T1Cu_C_ and T1Cu_N_ sites in *Ts*NirK. Spin-polarized WB97XD functional including empirical dispersion (Chai & Head-Gordon, 2008 ▸) and an Amber classical force field (Cornell *et al.*, 1995 ▸) were used for the QM and MM computations, respectively. The residues included in the QM part for the T1Cu_N_ site were Gln130, Cys115, His125 and His75, meanwhile for T1Cu_C_ site the residues were His431, His390, Cys428, Met434 and Arg389. Both copper atoms have a charge of 2+ and the His residues are protonated on the non-copper-bonded nitrogen. The forces on the atoms were relaxed before computing and the Raman spectra.

## 3. Results   

### 3.1. Phylogeny of *Ts*NirK   

**Figure 1:**
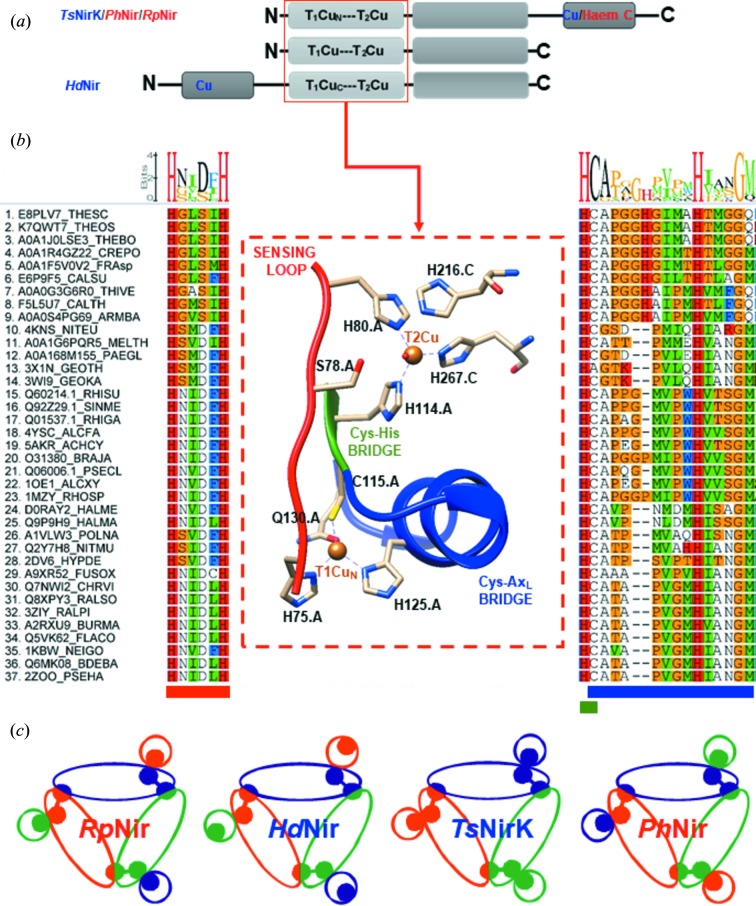
Structure-based sequence alignment of the T2Cu–T1Cu_N_ amino-acid region of *Ts*NirK. (*a*) Diagrammatic representation of polypeptide sequence and domain distribution in NirKs. (*b*) Alignment of the sequences in the T1Cu–T2Cu core region of 37 NirKs (putative or well characterized) performed with *Geneious* 7.0. A detailed protein-source nomenclature is shown in Fig. S1. Sequence alignment corresponding to the sensing-loop region (on the left in red) and alignment of the amino-acid sequence that connects the Cys with the axial ligand (Ax_L_) (on the right in blue). In the centre, a cartoon shows structural detail of regions in *Ts*NirK. (*c*) A cartoon arrangement for *Rp*NiR, *Hd*Nir, *Ts*NirK and *Ph*Nir. Metal cofactors are shown as dots and coloured according to their domains. The additional fused domains (cupredoxin/cytochrome) are shown as circles.

*BLAST* and protein-sequence-alignment analysis show an N-terminal region typical of two-domain NirKs, and also a C-terminal extension [Fig. 1 ▸(*a*)]. This extra domain belongs to the cupredoxin superfamily and shares ∼30% identity with several monomeric cupredoxins (see Table S1 in the Supporting information) involved in electron-transfer processes (Pérez-Henarejos *et al.*, 2015 ▸). A set of 37 NirK sequences was selected for alignment, including similar elongated NirK proteins as well as several well characterized NirKs with reported crystal structures [T1Cu–T2Cu core complex sequence, Fig. 1 ▸(*b*)]. The bootstrap consensus tree (see Fig. S1) groups *Ts*NirK within a cluster with uncharacterized putative NirKs, all with extended C-terminal sequences. These sequences not only come from closely related microorganisms of the *Thermus* genus that share *ca* 80% sequence identity, *e.g. T. brockianus* and *T. oshimai* JL-2, but also from unrelated microorganisms that share only *ca* 50% sequence identity, *e.g. Fraserbacteria sp.* (408 amino acids; 59%) and the methane oxidizer *Crenothrix polyspora* (436 amino acids; 55%). The latter allow us to infer that *Ts*NirK belongs to a new subgroup within class III NirKs (Ellis *et al.*, 2007 ▸; Horrell *et al.*, 2017 ▸).

### 3.2. Overall structure   

**Figure 2:**
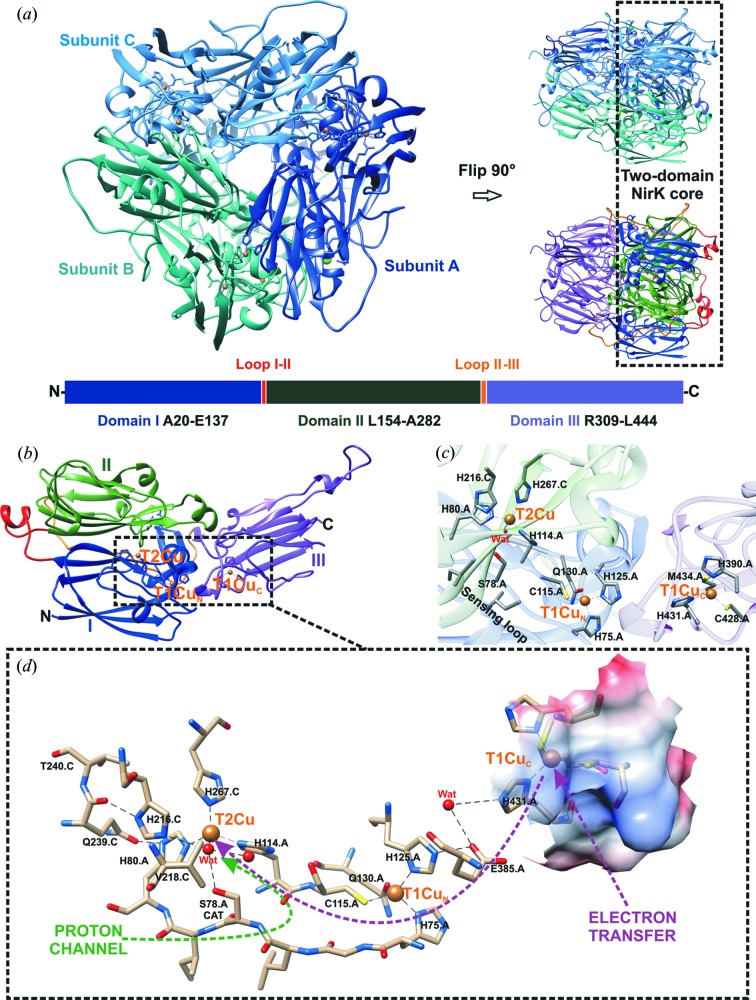
Structural organization of *Ts*NirK. (*a*) Homotrimer viewed from different angles. The two-domain NirK core structure is attached to the extra C-terminal domain arrangement (right panel). A diagram of the distribution of domains along the sequence is shown at the bottom. (*b*) Ribbon diagram of a monomer with domain I (blue) containing T1Cu_N_ and T2Cu, domain II (green), domain III (purple) harbouring the T1Cu_C_ centre, the linker loop (red) between domains I and II and the long loop (orange) between domain II and III. (*c*) Distribution and coordination spheres of each copper centre. The representation of T1Cu_N_–T2Cu connections: Cys115–His114 bridge and the Ser78_CAT_-containing sensing loop (His75 to His80). (*d*) Proton-channel electron-transfer-coupled pathway. The coulombic coloured surface shows the possible structural contact area with physiological mediators.

Expression and purification to electrophoretic purity of the product of the optimized *Ts*NirK gene yielded a blue-coloured protein arranged in a homotrimeric complex (∼50 kDa subunits), as determined by size exclusion chromatography and SDS–PAGE (see Fig. S2). The copper content obtained was 3.2 ± 0.4 mol Cu per mol monomer. To investigate the overall structure and the interaction of the C-terminal domain fused to the two-domain core [Fig. 1 ▸(*a*)], as well as the unique features observed within the sequence alignment [Fig. 1 ▸(*b*)], *Ts*NirK was crystallized and its structure solved. Various precipitants from our initial screening yielded characteristic blue single crystals with good diffraction (<1.5 Å) within less than a week. Unfortunately, most of these crystals displayed substantial merohedral twinning, resulting in the trigonal space group *H*3 incorrectly being indexed as *H*32. All of these crystals contained a single protomer in the asymmetric unit cell (ASU), with the homotrimeric structure obtained through the threefold crystallographic symmetry axis (data not shown). However, a single crystal was obtained that indexed to *C*121, containing the entire homotrimer in the ASU. The structure was determined at 1.63 Å through molecular replacement using *Gt*Nir (PDB entry 3x1e), which shares 36% identity (57% homology) to the N-terminal region of *Ts*NirK, with the extended C-terminus built in the resulting observed density. Data collection and refinement statistics are shown in Table 1 ▸. The three monomers of *Ts*NirK’s biological unit are nearly identical, apart from small surface side-chain rotamers, and are related via a threefold non-crystallographic symmetry axis [Fig. 2 ▸(*a*), left panel]. Main-chain differences and weak density (high *B* factors) were also observed within the linker region between domain II and III, suggesting a high degree of flexibility.

The biological unit of *Ts*NirK is composed of three monomers [Fig. 2 ▸(*a*), left panel]. Three distinct domains can be distinguished within each monomer [Fig. 2 ▸(*a*), bottom, Fig. 2 ▸(*b*)]: domain I (Ala20–Glu137, N-terminal), domain II (Leu154–Ala282) and domain III (Arg309–Leu444, C-terminal). Domains I and III are located at the periphery of the trimer, while domain II, which is positioned in the core of the homotrimer structure, constitutes the inter-subunits inter­action domain [Fig. 2 ▸(*a*), right panel]. A linker loop connects domain I with domain II (Pro138–Asp153), whereas a second longer linker region (Lys283–Lys308) extends at the side of domain I connecting domain II and III, with domain III being on the top of the two-domain core structure [Fig. 2 ▸(*a*), right panel]. Domain III is closely attached to domain I by surface interactions. The characteristic extra loop (Asn181–Pro191) and the tower loop (Boulanger & Murphy, 2002 ▸; Fukuda, Koteishi *et al.*, 2014 ▸) (Tyr164–Leu176) of two-domain NirKs (Fukuda, Koteishi *et al.*, 2014 ▸) were observed within domain II. Domains I and III harbour the copper centres [Fig. 2 ▸(*b*)] and a calcium atom from the crystallization solution, coordinated by residues of domain I, is found between the monomers. Domain I harbours the characteristic T1Cu and T2Cu centres of two-domain NirKs, whereas domain III has an extra T1Cu centre.

### 3.3. Type 1 and type 2 Cu centres   

Coordination around T1Cu and T2Cu atoms is shown in Fig. 2 ▸(*c*). Domain I contains the T1Cu centre at the N-terminal region (T1Cu_N_) and the active T2Cu site [Fig. 2 ▸(*c*)]. Domain III contains a second T1Cu centre [T1Cu_C_, Fig. 2 ▸(*c*)]. Relevant distances and angles of T1Cu centres and their comparison with those observed in others NirKs and blue cupredoxins are shown in Table S2.

T1Cu_C_ is an amicyanin-like T1Cu centre (Holm *et al.*, 1996 ▸) with the His_2_ N^δ1^–Cys S^δ^–Met S^γ^ ligand set and an additional carbonyl O atom from Arg389 trans to the axial Met ligand (Pérez-Henarejos *et al.*, 2015 ▸). T1Cu_N_ is located at the top of domain I and is coordinated by two His N^δ1^ residues (His75 and His125), Cys115 S^δ^, and a Gln130 O^∊1^ residue in apical position. This copper site, which shows nearly tetrahedral coordination similar to that observed in stellacyanin (DeBeer George *et al.*, 2003 ▸), was never observed before in NirKs (Horrell *et al.*, 2017 ▸). The coordination sphere of the catalytic T2Cu is composed of three His N^∊2^ in a plane with the Cu atom and a water molecule in an apical position (1.96 ± 0.02 Å). His80 (2.02 ± 0.01 Å), and His114 (2.04 ± 0.03 Å) are provided by domain I, whereas His267 (2.06 ± 0.01 Å) comes from domain II of an adjacent subunit, as usually observed in NirKs.

The T1Cu_C_ centre is buried at ∼5 Å from the molecular surface of *Ts*NirK and is located ∼14.1 Å away from T1Cu_N_; T1Cu_N_ (proximal centre) and T1Cu_C_ (distal centre) are ∼12.6 Å and ∼22.3 Å away from T2Cu, respectively [Fig. 2 ▸ (*d*)]. T1Cu_C_ and T1Cu_N_ are linked by a chemical path that involves Glu385 and a water molecule [Fig. 2 ▸(*d*)]. T1Cu_N_ and T2Cu are connected by a typical Cys–His bridge (Cys115–His114) [Figs. 1 ▸(*b*) and 2 ▸(*d*)].

### 3.4. The Cys115–Gln130 structure region and its surrounding area is a key structural feature for the interaction of domain I and domain III   

The unique architecture of *Ts*NirK reveals that the loop with an α-helix (His120 to Thr126) located at domain I serves as a scaffold for several inter-domain interactions (see Fig. S3). The molecular surfaces of both domains involved in the interaction are complementary. Several hydrogen-bond interactions are clearly observed (see Fig. S3), with a number of water molecules in the contact region also reinforcing the hydrogen-bond network, contributing to the stabilization of inter-domain interactions. At least three interactions were observed in the surrounding area. These interactions take place in the contact region of a β-strand (Arg309 to Val311) at the N-terminus of domain III with a β-strand of the domain I (Val39 to Phe46): Arg309(O)–Tyr40(N), Val311(O)–Arg42(N) and Val311(N)–Tyr40(O).

### 3.5. An uncommon sensing loop connects T1Cu_N_ with T2Cu and configures a new active-site pocket   

As reported for all NirKs (Strange *et al.*, 1999 ▸), the connection between the electron-transfer T1Cu centre and the catalytic T2Cu site takes place via a Cys–His bridge and a His-*X*_4_-His substrate sensing loop [Figs. 1 ▸(*b*), 2 ▸(*c*) and 2 ▸(*d*)]. The sequence of *Ts*NirK reveals a unique amino-acid composition at the sensing loop that has not been observed before [Fig. 1 ▸(*b*)]. The His75-Gly76-Leu77-Ser78_CAT_-Ile79-His80 substrate sensing loop configures a novel active-site pocket in which the Asp_CAT_(COOH) is replaced by Ser_CAT_(CH_2_—OH), with Ser_CAT_ being in close proximity to the T2Cu centre bound water [Fig. 2 ▸(*d*)]. The active-site pocket of *Ts*NirK also harbours the residues Val218 (Val_CAT_), His216 (His_CAT_), Gln239 and Thr240, which have been proposed to be relevant in catalysis in two-domain NirKs. Furthermore, several water molecules connect Ser_CAT_ with His_CAT_ via Gln239 and Thr240 in a hydrogen-bond network [Fig. 2 ▸(*d*)].

### 3.6. The substrate access channel to the type 2 copper centre and the proton channel   

The T2Cu centre can be accessed through an ∼16 Å deep channel (see Fig. S4) that covers an area of 570 Å^2^. This substrate access channel is formed by amino acids of two adjacent subunits that are hydrogen-bonded to some of the water molecules in the channel. Part of the wall of this channel is formed by several hydrophobic residues that constitute a network surrounding the T2Cu site, similar to that observed in two-domain NirKs (Hough *et al.*, 2008 ▸; Leferink *et al.*, 2011 ▸; Horrell *et al.*, 2017 ▸). This network (Val218, Val265, and Ile121) is located along one side of the T2Cu and settles ∼6 Å from the active site. Only one putative proton channel is identified in the *Ts*NirK structure [Ser_CAT_–(4 × wat)–Ala116–(2 × wat)–Gly91–Asn92].

### 3.7. Functional and spectroscopic characterization of recombinant *Ts*NirK   

*Ts*NirK was able to reduce nitrite with an apparent turnover of 65 ± 1 s^−1^, an apparent *K*_M_ value of 27 ± 2 µ*M* NO_2_^−^, and a catalytic efficiency of 2.4 × 10^6^ *M* ^−1^ s^−1^ (see Fig. S5). Furthermore, the enzyme reoxidized a pseudoazurin from *Sinorhizobium meliloti* 2011 (*Sm*Paz) in the presence of nitrite in an argon-flushed septa-sealed cuvette showing the capacity for interaction with an external cupredoxin-like electron donor (see Fig. S6).

**Figure 3:**
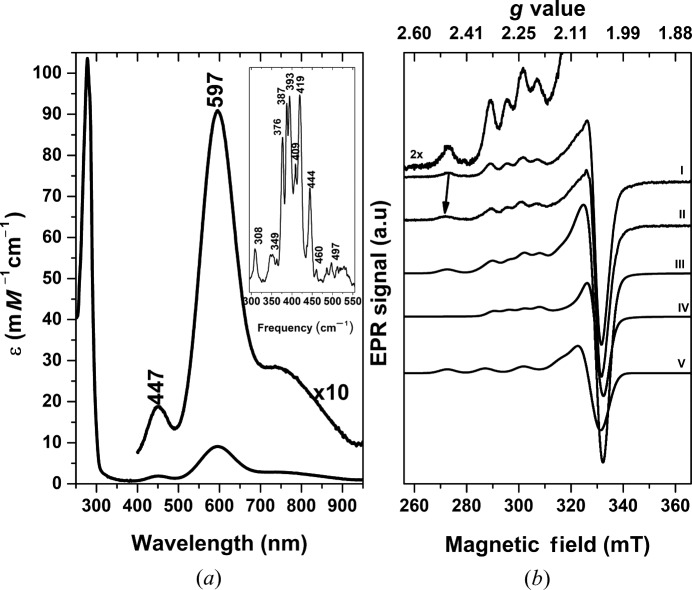
UV–Vis electronic absorption spectrum, rR spectrum, and EPR spectra of *Ts*NirK. (*a*) UV–Vis spectrum of as-purified *Ts*NirK in 20 m*M* Tris–HCl buffer (pH 7.0) at 298 K. The rR spectrum was acquired with an excitation laser at 631.9 nm at 77 K (inset). (*b*) EPR spectra of as-prepared enzyme (I), added of nitrite (II), and simulation (III). The T1Cu and T2Cu spectral components (IV and V, respectively) were combined assuming a T1Cu:T2Cu ratio of ∼2:1. T1Cu: *g*_1,2,3_ = 2.260, 2.054, 2.034 and *A*_1,2,3_ = 5.9, n.d., n.d.; T2Cu: *g*_1,2,3_ = 2.296, 2.076, 2.054 and *A*_1,2,3_ = 14.5, n.d., n.d. (where n.d. means non-detectable). All the EPR experiments were carried out at 120 K.

The UV–Vis spectrum is characteristic of a blue-copper-nitrite reductase with absorption bands at ∼447 nm [S(σ)_Cys_ → Cu LMCT band], ∼597 nm [S(π)_Cys_ → Cu LMCT band] and a shoulder within the 700–800 nm region (*d*–*d* transitions) [Fig. 3 ▸(*a*); Table S3] (Holm *et al.*, 1996 ▸; Zumft, 1997 ▸). Table S3 shows the UV–Vis spectroscopic features of *Ts*NirK and their comparison with those observed in other NirKs and blue cupredoxins. Reduction of *Ts*NirK with sodium dithionite under argon atmosphere led to the disappearance of the UV–Vis bands (not shown), in line with T1Cu centres in their reduced state. Reoxidation upon addition of nitrite under argon atmosphere partially recovered the as purified protein UV–Vis spectrum showing a slight blue shift of 5 nm of the band at 597 nm.

The 77 K resonance Raman (rR) spectra of *Ts*NirK excited at 631.9 nm show six intense main peaks in the range of 350 to 450 cm^−1^ together with several less intense resonances out of this range (see inset in Fig. 3 ▸). Reoxidation by nitrite addition to dithionite-reduced *Ts*NirK essentially recovered the rR spectrum of the as-purified enzyme. QM/MM calculations based on the solved crystal structure of *Ts*NirK showed an r.m.s. deviation for the QM-treated atoms of ∼0.1 Å and ∼0.15 Å for T1Cu_N_ and T1Cu_C_, respectively, in good agreement with the experimental structural data. The resulting T1Cu_N_ and T1Cu_C_ QM/MM models obtained were used to predict the corresponding Raman spectra. These calculations showed two distinguishable Cu–S(Cys) stretching resonances at 347 cm^−1^ and 414 cm^−1^ for the T1Cu_N_ and T1Cu_C_, respectively (see Fig. S7), suggesting that the main rR peaks observed in the range 350–450 cm^−1^ come from the two structurally characterized T1Cu centres.

EPR spectra at 120 K of as-purified *Ts*NirK [Fig. 3 ▸(*b*), spectrum I] show partially overlapped nearly axial components, all of them with a solved hyperfine structure at *g_||_*, typical of T1Cu and T2Cu centres in the Cu^2+^ oxidation state. EPR parameters are given in Table S3. Ferricyanide addition to as-purified *Ts*NirK did not significantly modify either the shape of the line or the intensity, suggesting that the three copper centres are all Cu^2+^ ions. No EPR signals were observed upon dithionite excess addition. EPR spectra also show the typical behaviour observed in two-domain NirKs upon nitrate addition, *i.e*. a slight shifting of the *g_||_* feature of the T2Cu EPR signal [Fig. 3 ▸(*b*), spectrum II], which is indicative of T2Cu–nitrite interaction.

## 4. Discussion   

*Ts*NirK is the fourth three-domain Nir crystallized so far among the copper nitrite reductases. The overall structure of *Ts*NirK [Fig. 2 ▸(*a*)] shows a unique distribution of domains and subunit interactions that differs greatly from those reported for *Hd*Nir (PDB entry 2dv6; Nojiri *et al.*, 2007 ▸), *Rp*Nir (PDB entry 3ziy; Antonyuk *et al.*, 2013 ▸) and *Ph*Nir (PDB entry 2zoo; Tsuda *et al.*, 2013 ▸) [Fig. 1 ▸(*c*)]. *Hd*Nir, *Rp*Nir and *Ph*Nir have an extra C-terminal or N-terminal domain harbouring a haem *c* or a T1Cu cofactor that does not interact with the two-domain core of the same subunit (Antonyuk *et al.*, 2013 ▸; Nojiri *et al.*, 2007 ▸; Tsuda *et al.*, 2013 ▸) [Fig. 1 ▸(*c*)]. This is not the case with *Ts*NirK, where the extra C-terminal domain interacts directly with the T1Cu–T2Cu complex of the same subunit [Fig. 1 ▸(*c*) and the right panel of Fig. 2 ▸(*a*)]. The distal T1Cu_C_ of *Ts*NirK is located at the C-terminal region, while that of *Hd*Nir is at the N-terminal region. Another remarkable difference is that the T1Cu_C_ centre of *Ts*NirK is ∼14 Å away from the proximal T1Cu, while the nearest distal T1Cu in *Hd*Nir is located at ∼24 Å (Nojiri *et al.*, 2007 ▸). The distal T1Cu centre of *Hd*Nir was demonstrated to be unable to shuttle electrons for nitrite reduction. Based solely on the structural characteristics of *Ts*NirK, the electron-transfer pathway T1Cu_C_ → T1Cu_N_ → T2Cu is highly probable in this enzyme, as is the case for *Rp*Nir where the haem *c* cofactor and the T1Cu centre are 10 Å apart (Antonyuk *et al.*, 2013 ▸).

The UV–Vis electronic absorption spectrum of *Ts*NirK [Fig. 3 ▸(*a*)] resembles those from blue cupredoxins with intensities and a band distribution similar to those observed in *Alcaligenes xylosoxydans* Nir (*Ax*Nir), *Cucumis sativus* stellacyanin (*Cs*Ste) and amicyanin (see Table S3). *Ts*NirK is intense blue (∊1/∊2 = 0.21) compared with the greenish–blue three-domain *Hd*Nir (∊1/∊2 = 0.46) (see Table S3). Addition of an excess of sodium nitrite to dithionite-reduced *Ts*NirK partially recovers the observed as-purified enzyme spectrum, which suggests that the two T1Cu centres are involved in electron transfer. The reoxidation is accompanied by a slight shift to a lower wavelength (5 nm) from the 597 nm band. This type of shift was also observed in the two-domain *Sm*Nir when subjected to anaerobic reoxidation in the presence of nitrite (Ferroni *et al.*, 2012 ▸). Whether this blue shift is a consequence of a dithionite presence in the medium or is a product of only one T1Cu centre being reoxidized upon nitrite addition cannot be elucidated with the present data.

Whereas UV–Vis and *X*-band EPR spectroscopies cannot discriminate between the two T1Cu centres of *Ts*NirK, more valuable information can be obtained by rR spectroscopy. The principal Raman spectral features of selected examples of T1Cu-containing proteins that resemble those present in *Ts*NirK are summarized in Fig. S8. As shown in this figure, the main resonance peaks of *Ts*NirK fall in the range of 350–450 cm^−1^, in agreement with cupredoxin rR spectra reported so far (Han *et al.*, 1991 ▸, 1993 ▸; Andrew *et al.*, 1994 ▸). For amicyanins (Sharma *et al.*, 1988 ▸; Buning *et al.*, 2000 ▸), which contain a T1Cu centre that resembles T1Cu_C_ of *Ts*NirK, the more intense peak falls in the region 410–430 cm^−1^ (see Fig. S8, yellow-shaded region). In contrast, for stellacyanins (Nersissian *et al.*, 1996 ▸; DeBeer George *et al.*, 2003 ▸) containing T1Cu centres resembling T1Cu_N_, the main resonance peak falls in the region 350–410 cm^−1^ (See Fig. S8, grey shaded area). Hence, this suggests that the *Ts*NirK rR spectrum is the superposition of two distinguishable Cu^2+^ T1Cu species, a conclusion also predicted by QM/MM calculations (see Fig. S7).

Nitrite reduction by NirKs can be divided into three main steps, the interaction between the enzyme and an external physiological electron donor, an internal electron-transfer reaction involving the copper centres, and nitrite-T2Cu interaction to release NO (Brenner *et al.*, 2009 ▸; Leferink *et al.*, 2011 ▸; Nojiri *et al.*, 2009 ▸).

The putative electron donor of *Ts*NirK is a cytochrome *c*_552_-like protein encoded by the *tsc*17520 gene located in the denitrification cluster in the *T. scotoductus* SA-01 genome (Gounder *et al.*, 2011 ▸). We do not discard the possibility that other mediators located far away from the denitrification cluster in *T. scotoductus* SA-01 can also act as electron donors as observed for *Bradyrhizobium japonicum* USDA 110 (Bueno *et al.*, 2008 ▸). Analysis of the domain III surface of *Ts*NirK reveals that the possible binding region for external electron donors is a pocket that covers the T1Cu_C_ site, which is determined by the hydrophobic Ile430 and the surrounding polar/charged residues Arg389, Asp391, Lys407 and Ser429 [Fig. 2 ▸(*d*)]. This pocket would allow transient interactions with external physiological electron donors like in other transient complexes (Kataoka *et al.*, 2003 ▸; Nojiri *et al.*, 2009 ▸; Tsuda *et al.*, 2013 ▸). Kinetic experiments (see Fig. S6), performed with *Ts*NirK and *Sm*Paz, the physiological partner of *Sm*Nir (Ferroni *et al.*, 2014 ▸), showed a rate ∼7 times slower than that of *Sm*Nir under the same reaction conditions, demonstrating that this enzyme can function with external electron donors from other sources. The only way for interaction between *Sm*Paz and *Ts*NirK might be the domain III crown [Fig. 2 ▸(*a*)]. The domain III crown seems to act like a compact structure [Fig. 2 ▸(*a*)] covering the T1Cu_N_ site located at the domain I–II NirK core structure. This constitutes a difference compared with *Ph*Nir, in which the extra domain can move apart from NirK core structure allowing the interaction of the external physiological electron donor either with T1Cu or with the tethering cytochrome *c* (Tsuda *et al.*, 2013 ▸)*.*

Structural data for *Ts*NirK suggest a potential electron-transfer pathway of T1Cu_C_ → T1Cu_N_ → T2Cu as there is no exposed hydrophobic patch through which a physiological external electron donor can potentially interact directly with the T1Cu_N_ centre. The most likely T1Cu_C_ → T1Cu_N_ electron-transfer route would involve domains I and III within the same subunit [Figs. 2 ▸(*d*) and S3]; in this pathway T1Cu_C_ might deliver electrons via a hydrogen-bonded His431 N^∊2^–wat–Glu385 O^∊1^–His125 N^∊2^ chemical path to the T1Cu_N_ centre. The water molecule involved in this putative electron-transfer pathway belongs to a hydrogen-bond network that also helps to stabilize domain I–domain III interaction (see Fig. S3). A similar water-molecule network is also observed in the contact area between the haem *c* domain and the surface above T1Cu of *Rp*Nir (Antonyuk *et al.*, 2013 ▸). This water network is not observed in the transient *Ax*Nir–Cyt *c*_551_ binary complex, where the interaction is mostly hydrophobic (Nojiri *et al.*, 2009 ▸). Several other amino acids in the contact surface are involved in the stabilization of the domain I–domain III complex of *Ts*NirK. For instance, the His120–Thr126 helix provides some of these amino acids which, as seen above, play a relevant role in the stabilization of the interdomain complex (see Fig. S3). The T1Cu_N_ → T2Cu electron-transfer pathway of *Ts*NirK consists of the well characterized Cys–His bridge observed in all NirKs reported so far (Brenner *et al.*, 2009 ▸; Cristaldi *et al.*, 2018 ▸; Leferink *et al.*, 2011 ▸; Strange *et al.*, 1999 ▸). Electron delivery towards the T2Cu active site through the Cys–His bridge has been demonstrated to be regulated by the so-called ‘sensing loop’, which harbours an Asp_CAT_ residue essential for catalysis (Boulanger *et al.*, 2000 ▸; Kataoka *et al.*, 2000 ▸; Strange *et al.*, 1999 ▸). A hallmark of *Ts*NirK architecture is a sensing loop harbouring a Ser78 (Ser_CAT_) residue instead of an Asp_CAT_ residue. This fact constitutes novelty from a catalytic perspective, reinforcing the idea that *Ts*NirK belongs to a new group of three-domain NirKs that should be classified separately from those described by Ellis *et al.* (2007 ▸).

**Figure 4:**
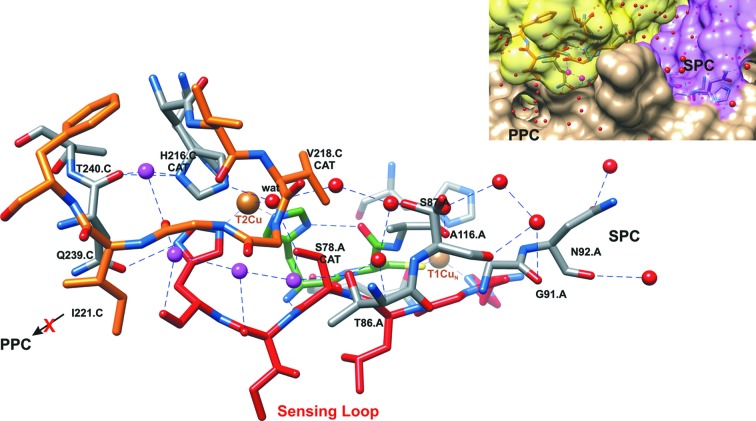
Hydrogen-bond network at the T2Cu active-site pocket. The primary proton channel (PPC) is blocked by Ile221 in *Ts*NirK and the secondary proton channel (SPC) is accessed from the bulk solution. The contribution of subunit A and the subunit C to build the external surface and the channel mouth are indicated in magenta and yellow, respectively (inset). Residues that build the sensing loop (red), the Cys–His bridge (green) and the amino-acid chain from Val_CAT_ to Phe222 (orange) are indicated. A hydrogen-bond network is built by several water molecules from the mouth of the channel to the T2Cu active site (in red) and a chain of occluded water molecules (in magenta). The occluded water molecules connect Ser_CAT_ to His_CAT_ via Gln239 and Thr240. Thr86 and Ser87 link the water chains. Ala116, Leu77, Gly91 and Asn92 are involved in the hydrogen-bond network towards the protein surface.

The T2Cu site of *Ts*NirK can react with nitrite in the Cu^2+^ oxidation state, as is evident from the EPR experiments [spectrum II in the right panel of Fig. 3 ▸(*b*)], with an apparent *K*_M_ value (27 µ*M* nitrite) only comparable to that of *Rp*Nir (Han *et al.*, 2012 ▸). This means that the enzyme can function at the highest possible rate at low nitrite concentrations, which is in agreement with the environmental conditions where *T. scotoductus* SA-01 grows (∼10^−7^–10^−6^ *M* nitrate) (Borgonie *et al.*, 2011 ▸; Magnabosco *et al.*, 2014 ▸). The process of nitrite reduction at the T2Cu active site requires the consumption of two protons, which has been intensively investigated in two-domain NirKs (Boulanger *et al.*, 2000 ▸; Hough *et al.*, 2008 ▸; Kataoka *et al.*, 2000 ▸; Leferink *et al.*, 2011 ▸). Two distinct proton channels, named primary and secondary, have been proposed to transport these protons, with the secondary channel being identified as the relevant one (Hough *et al.*, 2008 ▸). The putative proton channel in *Ts*NirK (Fig. 4 ▸), which shares some regions involved in the substrate channel and ends with the T2Cu bound water (see Figs. 4 ▸ and S4), resembles the secondary proton channel reported in *Alcaligenes xylosoxidans* Nir (*Ax*Nir) (Asp_CAT_–wat–wat–Ala131–Asn90–Asn107) (Hough *et al.*, 2008 ▸). The idea that *Ts*NirK has only one proton channel is also reinforced by the fact that the His residue that regulates the primary channel in two-domain NirKs (Hough *et al.*, 2008 ▸) (*Ax*Nir; *A faecalis* Nir, *Af*Nir; *Achromobacter cycloclastes* Nir, *Ac*Nir; *Rhodobacter sphaeroides* Nir, *Rs*Nir) is not found in *Ts*NirK (Fig. 4 ▸) or *Gt*Nir (Fukuda *et al.*, 2016 ▸) and three-domain NirKs (Nojiri *et al.*, 2007 ▸; Antonyuk *et al.*, 2013 ▸).

The T2Cu water ligand, which is linked to Asp_CAT_ in all NirKs reported so far, is bridging Ser_CAT_ and His_CAT_ residues in *Ts*NirK (Fig. 4 ▸). The second water molecule that bridges Asp_CAT_ and His_CAT_ in most NirKs (Boulanger & Murphy, 2001 ▸) is absent in *Ts*NirK. The T2Cu water ligand is replaced by nitrite during the catalytic cycle, and any modification of the T2Cu water ligand environment has implications in catalysis which is reflected in *k*_cat_ values (Boulanger & Murphy, 2001 ▸). The turnover of *Ts*NirK is higher than those reported for *Ax*Nir variants (D98A, D98E, and D98N) (Kataoka *et al.*, 2000 ▸) but 2.7 times less than for *Sm*Nir (Ferroni *et al.*, 2012 ▸), ∼7 times less than for *Ax*Nir (Kataoka *et al.*, 2000 ▸) and ∼12 times less than for the thermophilic *Gk*Nir (Fukuda, Koteishi *et al.*, 2014 ▸).

The *Ts*NirK crystal structure also shows additional residues postulated to be relevant for catalysis in two-domain NirKs, with Gln239 and Thr240 [Fig. 2 ▸(*d*)] catalytically equivalent to Glu279 and Thr280 in *Af*Nir (Boulanger *et al.*, 2000 ▸; Fukuda *et al.*, 2016 ▸) and in *Ac*Nir (Qin *et al.*, 2017 ▸). There is a hydrogen bond between His80 and the side chain of Gln239. His80 is located at the end of the sensor loop [Fig. 2 ▸(*d*)], which is thought to transmit information about the T2Cu status to T1Cu for electron delivery through the Cys–His bridge (Hough *et al.*, 2005 ▸; Strange *et al.*, 1999 ▸). The Thr240 is hydrogen-bonded to His_CAT_ [Fig. 2 ▸(*d*)]. An occluded water chain connecting Ser_CAT_ to His_CAT_ via a Gln239–Thr340 hydrogen-bond network, which is not observed in most two-domain NirKs, could also be relevant for *Ts*NirK functionality (Fig. 4 ▸). Another key residue is the highly conserved Ile_CAT_, which controls the mode of nitrite binding in NirKs (Boulanger & Murphy, 2009 ▸; Merkle & Lehnert, 2009 ▸). In *Ts*NirK, this residue is replaced by Val_CAT_, the same residue observed in *G. kaustophilus* Nir (*Gk*Nir) (Fukuda, Koteishi *et al.*, 2014 ▸) and *Gt*Nir (Fukuda, Tse *et al.*, 2014 ▸; Fukuda *et al.*, 2016 ▸).

In summary, all the structural properties of *Ts*NirK point to an enzyme that, despite having several of the essential catalytic features present in other NirKs, shows two distinctive and unique characteristics: firstly, the putative T1Cu_C_ → T1Cu_N_ → T2Cu electron-transfer pathway along the same subunit; and secondly, and more importantly, is the presence of the Ser_CAT_ residue at the enzyme substrate-sensing loop, which opens a new paradigm in this widely studied family of enzymes.

## 5. Related literature   

The following references are cited in the supporting information: Abraham *et al.* (1993) ▸; Nestor *et al.* (1984) ▸; Tocheva *et al.* (2007) ▸; Yamaguchi *et al.* (2004). ▸

## Supplementary Material

Supporting tables and figures. DOI: 10.1107/S2052252519000241/lz5022sup1.pdf

PDB reference: copper-nitrite reductase (NirK) 6hbe

## Figures and Tables

**Table 1:** Data collection and refinement statistics Values in parentheses are for the highest-resolution shell.

	*Ts*NirK (6hbe)
Data collection	
Space group	*C*121
Cell dimensions	
*a*, *b*, *c* (Å)	145.69, 110.55, 88.81
α, β, γ (°)	90.00, 107.51, 90.00
Resolution (Å)	86.51–1.63 (1.66–1.63)
*R*_merge_	0.049 (0.481)
〈*I*/σ(*I*)〉	11.8 (2.2)
Completeness (%)	98.3 (99.3)
Redundancy	3.4 (3.3)
	
Refinement	
Resolution (Å)	86.51–1.63
Number of reflections	155 447
*R*_work_/*R*_free_	0.163/0.190
Number of atoms	
Protein	10 047
Ligand/ion	12 (9Cu, 3Ca)
Water	853
*B* factors (Å^2^)	
Protein	29.02
Ligand/ion	24.74 (Cu), 23.53 (Ca)
Water	37.53
R.m.s. deviations	
Bond lengths (Å)	0.013
Bond angles (°)	1.488
