# Supplementary material for: A three-domain copper-nitrite reductase with a unique sensing loop
Source: IUCrJ. 2019 Feb 9;6(Pt 2):248–58. doi: 10.1107/S2052252519000241 (PMC6400189; doi:10.1107/S2052252519000241)

IUCrJ

Volume 6 (2019)

Supporting information for article:

A three-domain copper-nitrite reductase with a unique sensing loop

Diederik Johannes Opperman, Daniel Horacio Murgida, Sergio Daniel Dalosto, Carlos Dante Brondino and Felix Martín Ferroni

Table S1 Cupredoxins with reported crystal structures that resembles the C-terminal extra domain of *Ts*NirK.

Protein	PDB	Microorganism	Loop Cys-Met	% Identity
Domain III-*Ts*NirK		*Thermus scotoductus SA-01*	CSIHPYM	-
Amicyanin (*Pd*Ami)	1MDA	*Paracoccus denitrificans*	CTPHPFM	31
Pseudoazurin	3TU6	*Sinorhizobium meliloti* 2011	CAPHVGM	31
Amicyanin (*Pv*Ami)	1ID2	*Paracoccus versutus*	CTPHPFM	30
Pseudoazurin	1BQK	*Achromobacter cycloclastes*	CTPHYGM	25
Plastocyanin	1BXU	*Synechococcus sp.*	CEPHRGAGM	25
Plastocyanin	1JXD	*Synechocystis* PCC6803	CEPHRGAGM	21
Azurin	1RKR	*Alcaligenes xylosoxidans*	CSFPGHFALM	19

Table S2 Geometric parameters of *Ts*NirK T1Cu centres and its comparison with other T1Cu.

Protein[a]	*Ts* NirK		*Hd* Nir		*Gk* Nir	*Ax* Nir	*Af* Nir	*Cs* Ste	*Pv* Ami
T1 Centres	C	N	C	N	-	-	-	-	-
Distances (Å)									
Cu–$His_1N^{\delta 1}$	2.02(0.01)[b]	2.05(0.01)	2.14	2.12	2.07	2.02	2.08	1.96	2.04
Cu–$CysS^{\gamma}$	2.27(0.02)	2.18(0.04)	2.13	2.23	2.13	2.20	2.22	2.18	2.13
Cu–$His_2N^{\delta 1}$	2.04(0.02)	2.04(0.01)	2.10	2.11	2.03	2.03	2.07	2.04	2.13
Cu–$MetS^{\delta}$	3.02(0.04)	–	2.55	2.90	2.61	2.45	2.45	–	2.84
Cu–$GlnO^{\varepsilon 1}$	–	2.03(0.01)	–	–	–	–	–	2.21	–
Cu-O	3.58(0.02)	–	–	3.23	–	–	–	–	3.87
Angles (°)									
$His_1N^{\delta 1}$–Cu–$His_2N^{\delta 1}$	106(1)	99(1)	95	100	99	101	96	101	104
$His_1N^{\delta 1}$–Cu–$CysS^{\gamma}$	127,7(0.4)	121.5(0.5)	144	135	139	122	128	134	132
$His_1N^{\delta 1}$–Cu–$MetS^{\delta}$	80(1)	–	81	82	82	88	90	–	84
His_2 $N^{\delta 1}$–Cu–$CysS^{\gamma}$	123(1)	126.3(0.8)	104	120	103	114	107	118	111
$His_2N^{\delta 1}$–Cu– $MetS^{\delta}$	98(3)	–	116	97	117	116	130	–	101
$MetS^{\delta}$–Cu–$CysS^{\gamma}$	108.5(0.8)	–	116	111	113	114	107	–	117
$His_1N^{\delta 1}$–Cu–$GlnO^{\varepsilon 1}$	–	103.4(0.9)	–	–	–	–	–	94	–
$His_2N^{\delta 1}$–Cu–$Gln^{O\varepsilon 1}$	–	101(1)	–	–	–	–	–	102	–
$GlnO^{\varepsilon 1}$–Cu–$CysS^{\gamma}$	–	95.4(0.7)	–	–	–	–	–	101	–
Dihedral angle (φ)[c]	81.5	89.3	64.1	79.4	64.7	74.3	65.8	83.5	79.3

[a]The structures of the T1Cu sites of Nir from Hyphomicrobium denitrificans (HdNir), Geobacillus. kaustophilus (GkNir), Alcaligenes. xylosoxidans (AxNir), A. faecalis (AfNir), Cucumis sativus stellacyanin (CsSte), Paracoccus versutus amicyanin (PvAmi) are taken from PDB entries 2DV6, 3WI9, 1OE1, 1SNR, 1JER and 1ID2, respectively. His_1 and His_2 are the first and second His ligands in the amino acid sequence. TsNirK ligand distances and bond angles are an average of all three monomers. [b]Values in parentheses represents the standard deviation of measurements taken from the three monomers. [c]The dihedral angle (φ) is the angle between the planes $His_1N^{\delta 1}$-Cu-$His_2N^{\delta 1}$ and L_{axial}-Cu-$CysS^{\gamma}$.

Table S3 Spectroscopic features of NirK and its comparison with other NirKs, stellacyanin and amicyanin.

Protein[a]	*Ts*NirK	*Hd*Nir	*Ax*Nir	*Af*Nir	*Cs*Ste	*Pv*Ami
Type[b]	B	GB	B	G	B	B
Uv-Vis						
λ_1 (nm)	447	454	460	457	450	460
ε_1 ($mM^{-1}cm^{-1}$)	1.86	2.9	1.6	6.98	1.1	0.43
λ_2 (nm)	597	605	593	587	608	596
ε_2 ($mM^{-1}cm^{-1}$)	9.09	6.30	6.30	5.42	4.08	3.90
$\varepsilon_1/\varepsilon_2$	0.21	0.46	0.25	1.29	0.27	0.11
EPR (X-band)						
T1Cu [$g_{//}$; $A_{//}$ (mT)]	2.260; 5.9	2.21; 5.5[c]<br>2.23; 6.0[d]	2.208; 6.3	2.254; 7.4	2.290; 3.2	2.239; 5.6
T2Cu [$g_{//}$; $A_{//}$ (mT)]	2.296; 14.5	2.35; 13.5	2.298; 14.2	2.394; 13.0	–	–

[a]Copper nitrite reductases from *Hyphomicrobium denitrificans* (*Hd*Nir) (Yamaguchi *et al.*, 2004), *Alcaligenes xylosoxidans* (*Ax*Nir) (Abraham *et al.*, 1993), *A. faecalis* (*Af*Nir) (Tocheva *et al.*, 2007), *Cucumis sativus* stellacyanin (*Cs*Ste) (DeBeer George *et al.*, 2003) and *Paracoccus versutus* amicyanin (*Pv*Ami) (Buning *et al.*, 2000) are compared with *Ts*NirK. [b] color of the protein: B, Blue; GB, greenish-blue; G, green. [c,d]These values correspond to the values reported for C114A and C260A variants of *Hd*Nir, respectively (Yamaguchi *et al.*, 2004).

Figure S1 Molecular phylogenetic analysis by maximum likehood method. The unrouted phylogenetic tree shows de distribution of classical NirKs and novel three-domain NirK. The proteins are named by UniProt code or PDB (crystal structure available) followed by a 5 letter code identifying the source. THESC: *Thermus scotoductus* SA-01, THEBO: *T. brockianus*, THEOS: *T. oshimai* JL-2, CREPO: *Crenothrix polyspora*, FRAsp: *Fraserbacteria sp.*, CALSU: *Caldiarchaeum subterraneum*, THIVE: *Thioalkalivibrio versutus*, CALTH: *Caldalkalibacillus thermarum*, ARMBA: *Armatimonadetes bacterium* GSX, NITEU: *Nitrosomonas europaea*, MELTH: *Melghirimyces thermohalophilus*, PAEGL: *Paenibacillus glacialis*, GEOTH: *Geobacillus thermophilus*, GEOKA: *G. kaustophilus*, RHISU: *Rhizobium sullae*, SINME: Sinorhizobium meliloti 2011, RHIGA: R. galagae, ALCFA: *Alcaligenes faecalis*, ACHCY: *Achromobacter cycloclastes*, BRAJA: *Bradyrhizobium japonicum* USDA110, PSECL: *Pseudomonas chlororaphis*, ALCXY: *A. xylosoxidans*, RHOSP: *Rhodobacter sphaeroides*, HALME: *Halerofax mediterranei*, HALMA: *Haloarcula marismortui*, POLNA: *Polaromonas naphtalenivorans*, NITMU: *Nitrospira multiformis* ATCC 25195, HYPDE: *Hyphomicrobium denitrificans* A3151, FUSOX: *Fusarium oxysporum*, CHRVI: *Chromobacterium violaceum*, RALSO: *Ralstonia solanaceum*, RALPI: *R. picketii*, BURMA: *Burkholderia mallei* NTCT 10229, FLACO: *Flavobacterium columnare*, NEIGO: *Neisseria gonorrhoeae*, BDEBA: *Bdelvibrio bacteriovorus*, PSEHA: *Pseudoalteromonas haloplanctis*.

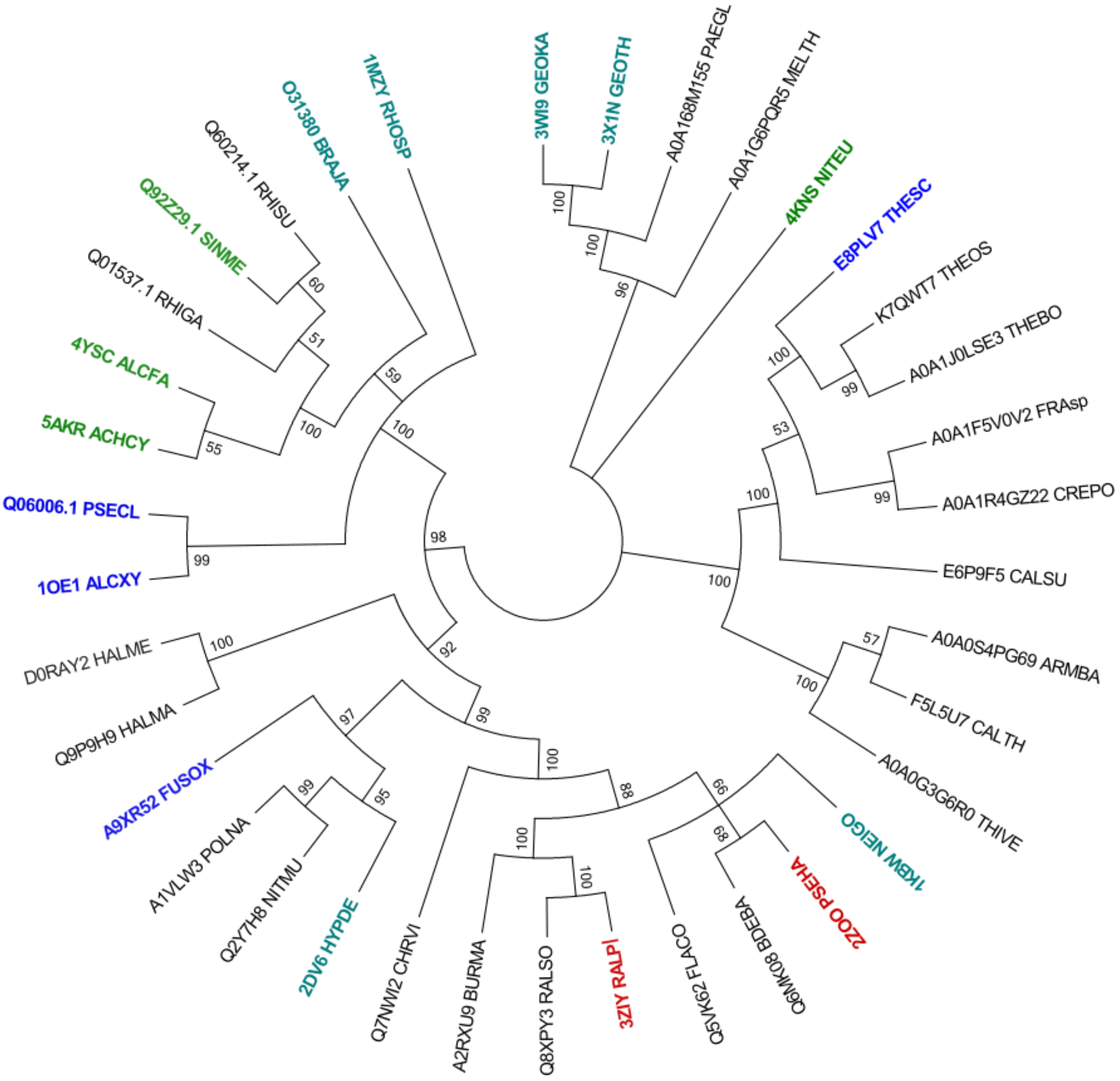
3WI9 GEOKA
3X1N GEOTH
A0A168M155 PAEGL
A0A1G6PQR5 MELTH
4KNS NITEU
E8PLV7 THESC
K7QWT7 THEOS
A0A1J0LSE3 THEBO
A0A1F5V0V2 FRAsp
A0A1R4GZ22 CREPO
E6P9F5 CALSU
A0A0S4PG69 ARMBA
F5L5U7 CALTH
A0A0G3G6R0 THIVE
1KBW NEIGO
2ZOO PSEHA
Q6MK08 BDEBA
Q5VK62 FLACO
3ZIY RALPI
Q8XPY3 RALSO
A2RXU9 BURMA
Q7NWI2 CHRVI
2DV6 HYPDE
Q2Y7H8 NITMU
A1VLW3 POLNA
A9XR52 FUSOX
Q9P9H9 HALMA
D0RAY2 HALME
1OE1 ALCXY
Q06006.1 PSECL
5AKR ACHCY
4YSC ALCFA
Q01537.1 RHIGA
Q92Z29.1 SINME
Q60214.1 RHISU
O31380 BRAJA
1MZY RHOSP

Figure S2 Size exclusion chromatography of TsNirK. A prepacked SuperdexTM 200 10/300 G2 column (GE Healthcare) connected to an Akta prime (GE Healthcare) system was equilibrated with 200 mM NaCl 20 mM Tris–HCl buffer (pH 7.6). Isocratic elution at a flow rate of 0.5 mL min-1 was performed with detection at 280 nm. The molecular weight markers used for calibration were ferritin (440 kDa), aldolase (158 kDa), conalbumin (75 kDa), ovalbumin (44 kDa) and carbonic anhydrase (29 kDa) and ribonuclease A (13.7 kDa). SDS-PAGE gel is shown. Prestained mid-range protein marker (2-105 kDa, Genbiotech) was used as protein ladder.

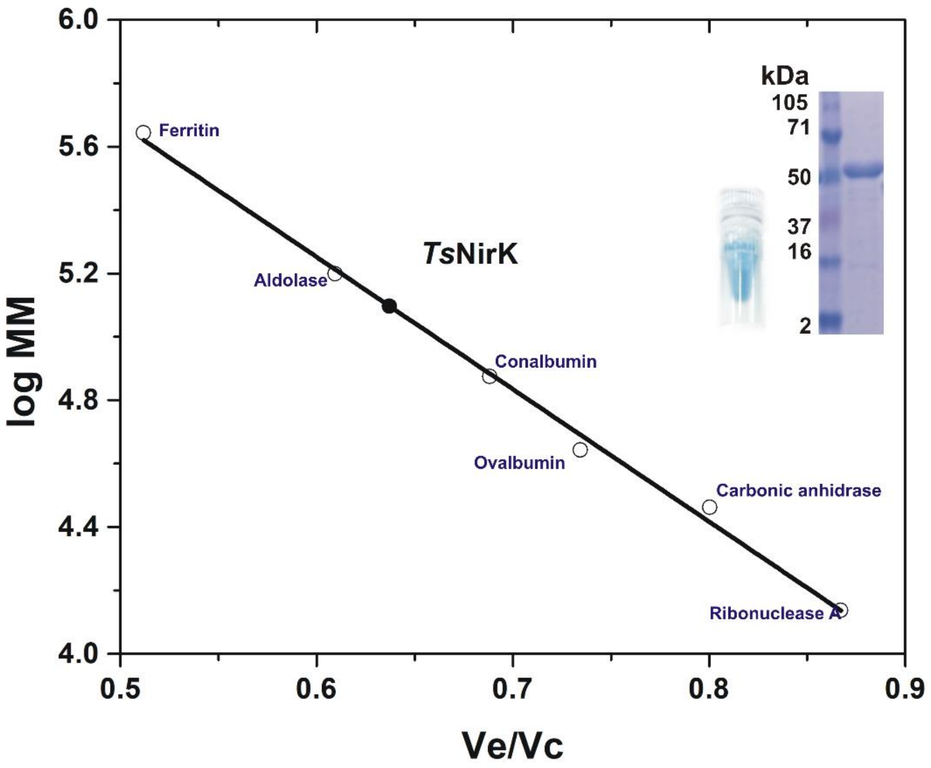

Figure S3 Domain I-domain III Interaction. Several amino acids of domain I (blue) and domain III (purple) are involved in the contact area. These amino acids interact trough H-bonds or salt bridges (black dashed lines). Also, a few amino acids from domain III interacts with amino acids within domain II of neighbouring subunit (II.c, in grey). A number of water molecules (red spheres) take part in the interdomain interaction. The possible $T1Cu_C \rightarrow T1Cu_N$ electron transfer pathway is indicated in magenta involving His431, Glu385, His125 and a water molecule. His120 ($N^{\varepsilon 2}$)–Asn356 ($O^{\varepsilon 1}$), Ala124 (O)–Tyr314 (OH) and His125 ($N^{\varepsilon 2}$)–Glu385 ($O^{\varepsilon 1}$).

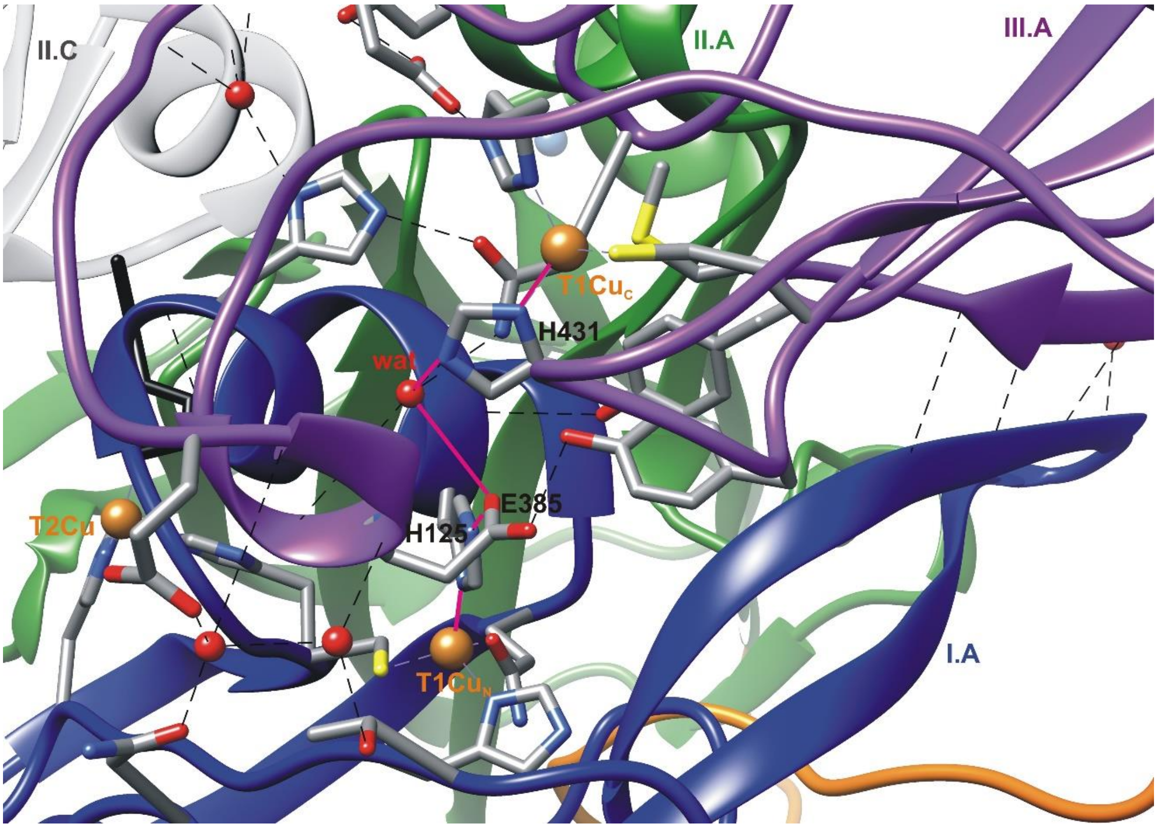

Figure S4 Details of the water channels in *Ts*NirK. (a, b). Three channels allow the entrance of the substrate to the T2Cu active site pocket located at the region delimited by domains I and II. **(c).** These channels are located at the contact surface space between domains I and II of adjacent subunits (I.A-II.C; I.C-II.B; II.A-I.B). **(d).** Subunit A provides (purple): Leu77, Ser78, Thr86, Ser87, Gln91, Asn92, His114, Ala116 to Gly19, Ile122, Met123, Phe379, Leu382 and Arg386. Subunit C (yellow): Val188, His216, Val218, Asp261, Thr263, Val265, His267, Phe269, Leu277, Ile279 and Arg281. A network of H-bonded water molecules connects the mouth of the channel with the catalytic active site. The substrate sensing loop is located underneath the contact surface of subunit A and some of the residues interact with the water molecule network: Leu77, Ser78, Thr86, Ser87, Ala116, Gly119, Asp261 and Thr263. A hydrophobic lining spreads from the mouth of the channel trough Leu277, Ile279, Val188, Phe379, Leu381 and Leu382.

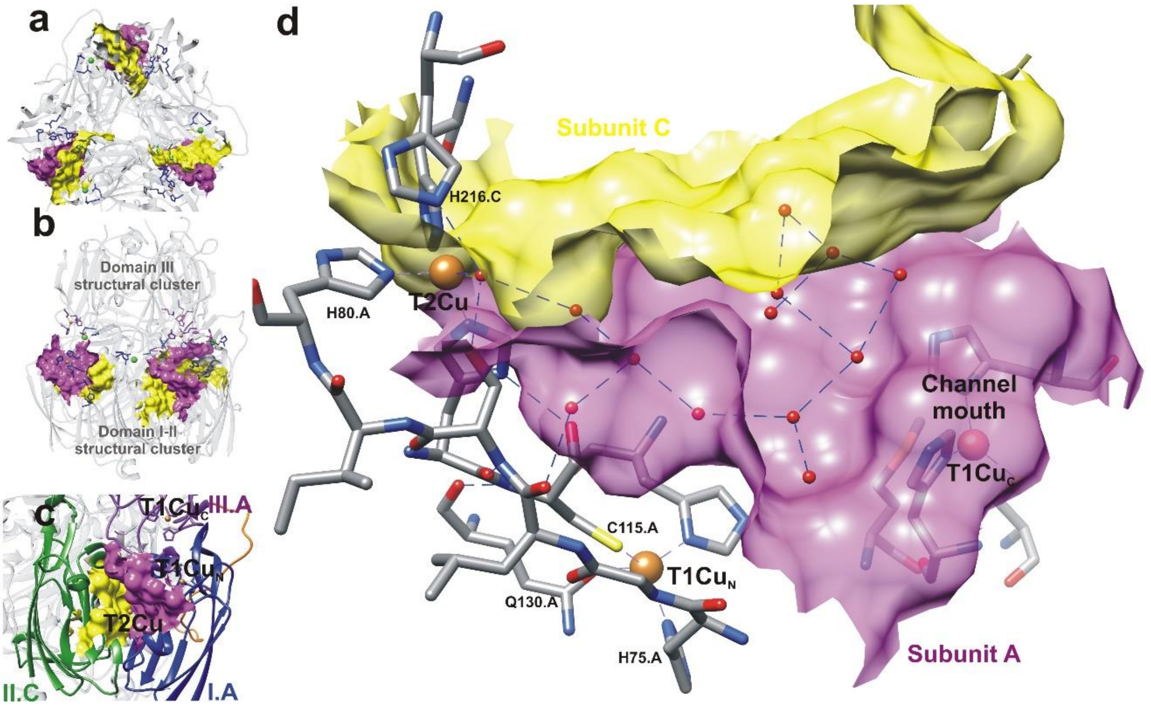

Figure S5 Plot of initial velocities (*v*) of Nir activities for the *Ts*NirK vs nitrite concentrations. The solid line shown the fitted curved based on the standard Michaelis-Menten model. All the reactions were performed in mixtures containing 5.4 nM *Ts*NirK (trimer).

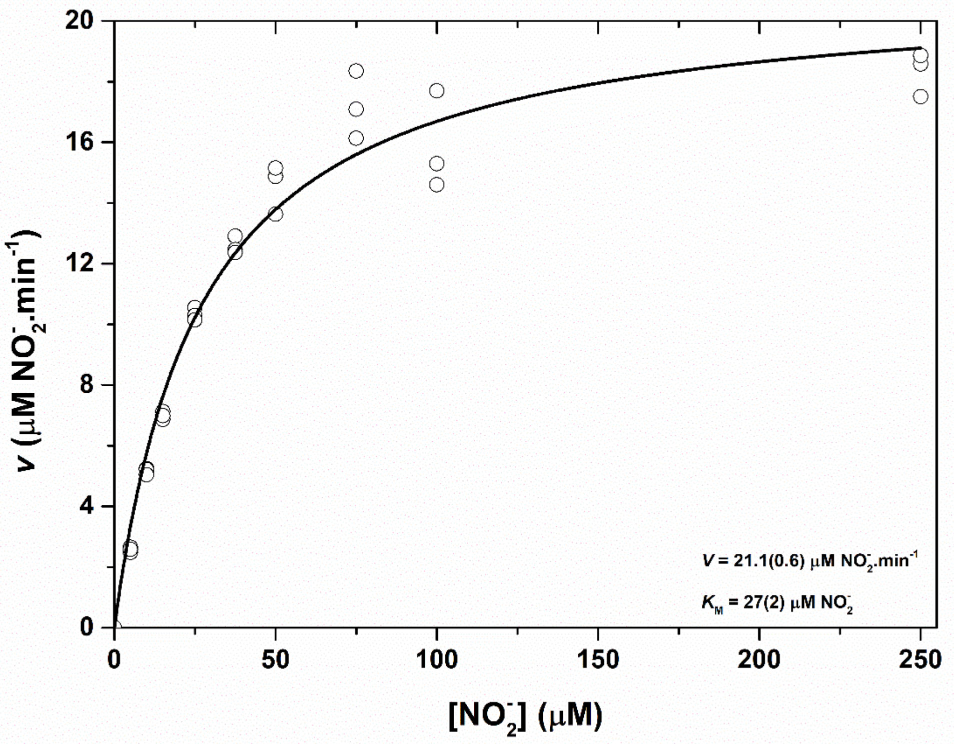

Figure S6 Time course of the absorbance changes at wavelength 597 nm in Nir assay using *Sm*Paz. The reoxidation of *Sm*Paz was followed at 597 nm. The mixture enzyme-*Sm*Paz reduced with sodium dithionite was mantained under argon flux during 2 min then the reaction was started by addition of argon-flushed sodium nitrite solution. The green and blue lines are *Sm*Nir (v_i = 0.55 ± 0.01 μM.s^{-1}) and *Ts*NirK (v_i = 0.075 ± 0.009 μM.s^{-1}) reactions, respectively. The black line corresponds to the reaction mixture with no enzyme as a reaction control. The measurements were performed in triplicates.

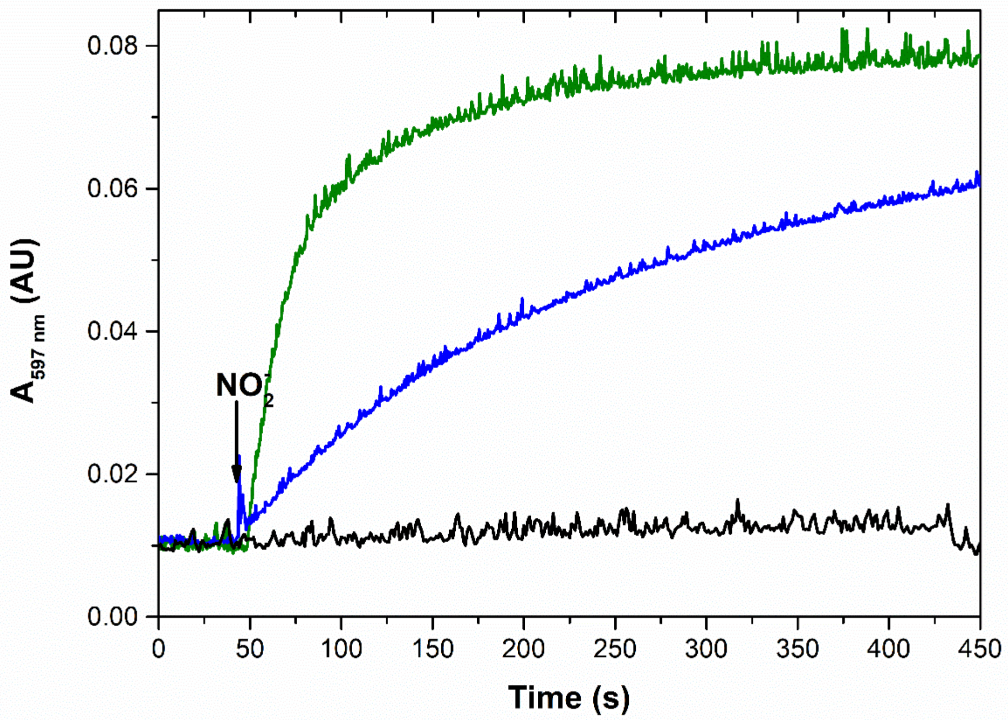

Figure S7 rR spectra calculated using QM/MM. The combination of Quantum Mechanics and Molecular Mechanics (QM/MM) calculations were used to compute the structure and the Raman spectra of the $T1Cu_C$ and $T1Cu_N$ sites in *Ts*NirK. The main Cu–S (Cys) vibrational modes are indicated for each T1Cu centre.

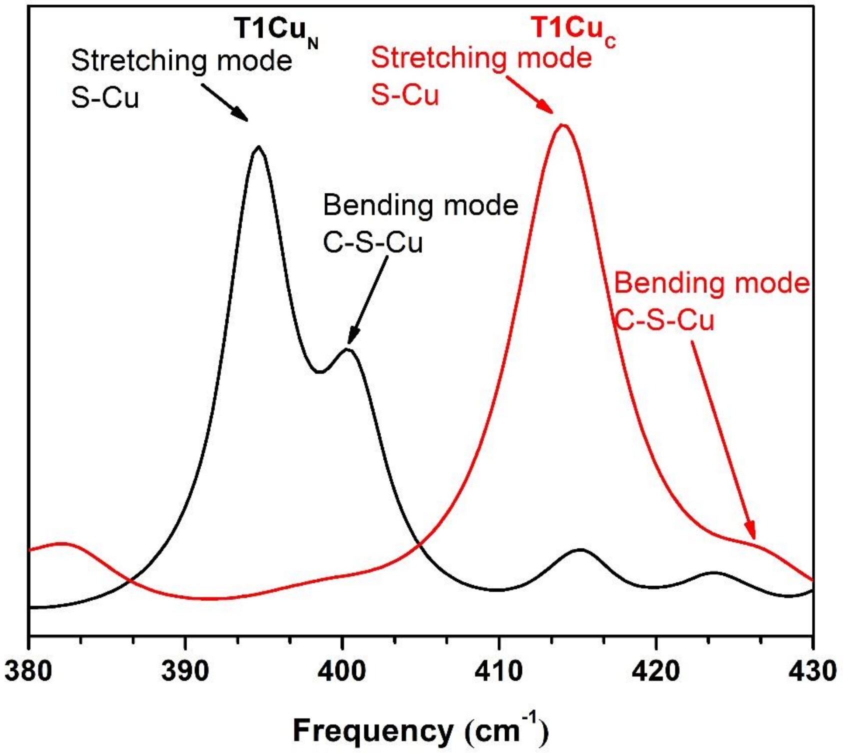

Figure S8 Relative peak intensities in the rR spectra of *Ts*NirK and related copper proteins. Data taken from sources detailed in text. *Rhus vernicifera* stellacyanin (*Rv*Ste) (Nestor *et al.*, 1984), *Cucumis sativus* stellacyanin (*Cs*Ste) (Nersissian *et al.*, 1996), *Paracoccus denitrificans* amicyanin (*Pd*Ami) (Sharma *et al.*, 1988), *P. versutus* amicyanin (*Pv*Ami) (Buning *et al.*, 2000). Inserts in each panel shows the loop sequence that connects Cys with the axial ligand (Met or Gln) in T1Cu centres. Gray area represents the υ(Cu–S) frequencies range for rhombic geometries and in yellow the axial geometry region (Andrew *et al.*, 1994).

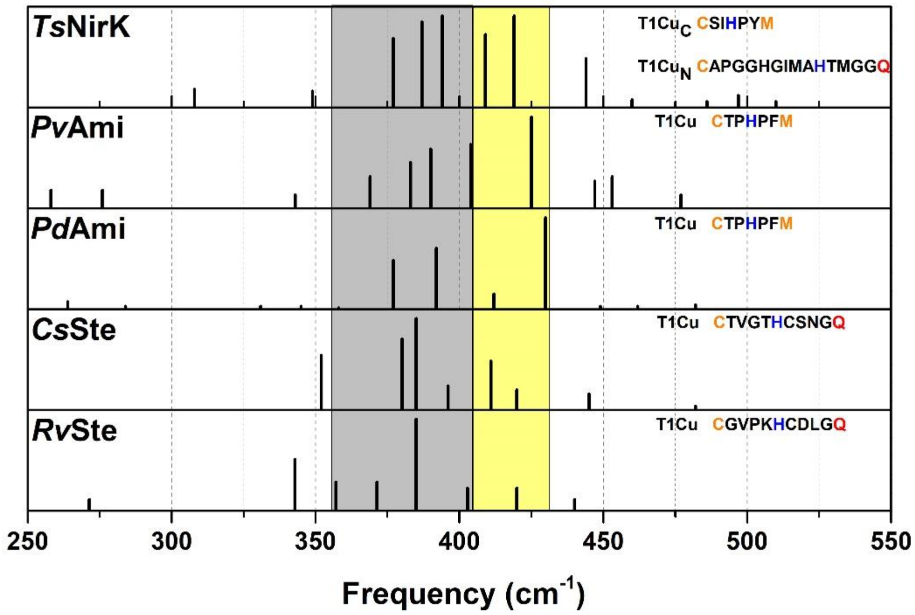

Supplement: Supplementary file 1 [file m-06-00248-sup1.pdf]
